# Association of Cardiorespiratory Fitness With Long-term Mortality Among Adults Undergoing Exercise Treadmill Testing

**DOI:** 10.1001/jamanetworkopen.2018.3605

**Published:** 2018-10-19

**Authors:** Kyle Mandsager, Serge Harb, Paul Cremer, Dermot Phelan, Steven E. Nissen, Wael Jaber

**Affiliations:** 1 Cleveland Clinic Foundation, Cleveland, Ohio

## Abstract

**Importance:**

Adverse cardiovascular findings associated with habitual vigorous exercise have raised new questions regarding the benefits of exercise and fitness.

**Objective:**

To assess the association of all-cause mortality and cardiorespiratory fitness in patients undergoing exercise treadmill testing.

**Design, Setting, and Participants:**

This retrospective cohort study enrolled patients at a tertiary care academic medical center from January 1, 1991, to December 31, 2014, with a median follow-up of 8.4 years. Data analysis was performed from April 19 to July 17, 2018. Consecutive adult patients referred for symptom-limited exercise treadmill testing were stratified by age- and sex-matched cardiorespiratory fitness into performance groups: low (<25th percentile), below average (25th-49th percentile), above average (50th-74th percentile), high (75th-97.6th percentile), and elite (≥97.7th percentile).

**Exposures:**

Cardiorespiratory fitness, as quantified by peak estimated metabolic equivalents on treadmill testing.

**Main Outcomes and Measures:**

All-cause mortality.

**Results:**

The study population included 122 007 patients (mean [SD] age, 53.4 [12.6] years; 72 173 [59.2%] male). Death occurred in 13 637 patients during 1.1 million person-years of observation. Risk-adjusted all-cause mortality was inversely proportional to cardiorespiratory fitness and was lowest in elite performers (elite vs low: adjusted hazard ratio [HR], 0.20; 95% CI, 0.16-0.24; *P* < .001; elite vs high: adjusted HR, 0.77; 95% CI, 0.63-0.95; *P* = .02). The increase in all-cause mortality associated with reduced cardiorespiratory fitness (low vs elite: adjusted HR, 5.04; 95% CI, 4.10-6.20; *P* < .001; below average vs above average: adjusted HR, 1.41; 95% CI, 1.34-1.49; *P* < .001) was comparable to or greater than traditional clinical risk factors (coronary artery disease: adjusted HR, 1.29; 95% CI, 1.24-1.35; *P* < .001; smoking: adjusted HR, 1.41; 95% CI, 1.36-1.46; *P* < .001; diabetes: adjusted HR, 1.40; 95% CI, 1.34-1.46; *P* < .001). In subgroup analysis, the benefit of elite over high performance was present in patients 70 years or older (adjusted HR, 0.71; 95% CI, 0.52-0.98; *P* = .04) and patients with hypertension (adjusted HR, 0.70; 95% CI, 0.50-0.99; *P* = .05). Extreme cardiorespiratory fitness (≥2 SDs above the mean for age and sex) was associated with the lowest risk-adjusted all-cause mortality compared with all other performance groups.

**Conclusions and Relevance:**

Cardiorespiratory fitness is inversely associated with long-term mortality with no observed upper limit of benefit. Extremely high aerobic fitness was associated with the greatest survival and was associated with benefit in older patients and those with hypertension. Cardiorespiratory fitness is a modifiable indicator of long-term mortality, and health care professionals should encourage patients to achieve and maintain high levels of fitness.

## Key Points

**Question:**

What is the association between cardiorespiratory fitness and long-term mortality?

**Findings:**

In this cohort study of 122 007 consecutive patients undergoing exercise treadmill testing, cardiorespiratory fitness was inversely associated with all-cause mortality without an observed upper limit of benefit. Extreme cardiorespiratory fitness (≥2 SDs above the mean for age and sex) was associated with the lowest risk-adjusted all-cause mortality compared with all other performance groups.

**Meaning:**

Cardiorespiratory fitness is a modifiable indicator of long-term mortality, and health care professionals should encourage patients to achieve and maintain high levels of fitness.

## Teaser

This study assesses the association between cardiorespiratory fitness and long-term mortality in adults undergoing exercise treadmill testing.

## Introduction

The inverse association between cardiorespiratory fitness (CRF) and mortality has been well established and is independent of age,^1,2,3^ sex,^4,5,6^ race/ethnicity,^7,8^ and comorbidities.^9,10,11,12,13^ Increased CRF is also associated with numerous cardiovascular and noncardiovascular benefits, including reductions in coronary artery disease (CAD),^14^ hypertension,^15^ diabetes,^16^ stroke,^17^ and cancer.^18^

However, recent observational studies^19,20,21,22^ have described adverse cardiovascular findings associated with habitual vigorous exercise and have raised new questions regarding the benefits of exercise and fitness. The hemodynamic stress of habitual vigorous exercise produces cardiovascular adaptations, including increases in cardiac chamber volumes, a balanced increase in left ventricular mass, and alterations in autonomic tone. Although these adaptations are usually thought of as physiologic and reversible, newer evidence has suggested associations between habitual vigorous exercise and potentially pathologic cardiovascular findings, including atrial fibrillation,^19^ coronary artery calcification,^20^ myocardial fibrosis,^21^ and aortic dilation.^22^ These findings have led some to propose a U-shaped dose-response association between exercise and cardiovascular events.^23^ In terms of mortality, a large pooled-cohort analysis of physical activity by Arem et al^24^ suggested a plateau effect of increasing physical activity volume. Other studies^25,26^ of self-reported jogging habits have revealed a dose-response plateau or even harm associated with the most strenuous jogging habits. However, studies^27^ linking physical activity levels with outcomes have relied on self-reported data and/or questionnaires; therefore, the inferences drawn from these studies are compromised by the limitations of recollection bias.

Exercise treadmill testing (ETT) is the most widely used method to measure CRF and serves as an objective measure of aerobic fitness and moderate-vigorous physical activity, without reliance on self-reported data. Previous studies^4,5,7^ of CRF and mortality have not specifically identified or analyzed patients with extremely high CRF, and it remains unclear whether there is an upper limit of CRF above which no further benefit or even harm is seen.

The present study assesses the association between aerobic fitness and all-cause mortality among the largest reported cohort, to our knowledge, of adult patients undergoing ETT at a tertiary care center. We additionally identified patients with extremely high CRF (elite performers; CRF ≥2 SDs above the mean for age and sex) to evaluate the relative benefit or harm of extreme CRF compared with more modest levels of aerobic fitness.

## Methods

### Study Design and Patient Population

We conducted a retrospective cohort study to evaluate the association between CRF, quantified by estimated metabolic equivalents (METs) on ETT, and all-cause mortality. The study population consisted of consecutive adult patients undergoing stress testing at our institution from January 1, 1991, through December 31, 2014. In cases of additional testing, only the first stress test was selected. Patients who underwent pharmacologic stress testing (n = 38 828) or who were converted to pharmacologic testing because of inability to reach 85% of maximum predicted heart rate (n = 467) were excluded. We excluded patients for whom sex information was missing (n = 796). Data analysis was performed from April 19 to July 17, 2018. The Cleveland Clinic Foundation Institutional Review Board approved the study and waived patient informed consent. Data were not deidentified.

At the time of stress testing, patient demographics (age and sex), anthropometrics (height, weight, and body mass index), medications, and comorbidities were prospectively documented. These comorbidities included history of CAD, diabetes, hypertension, hyperlipidemia, end-stage renal disease (ESRD), and smoking. Study definitions for comorbid conditions are provided in the eMethods in the Supplement. Data and analyses are presented in accordance with the Strengthening the Reporting of Observational Studies in Epidemiology (STROBE) reporting guideline for cohort studies.^28^

### ETTs and Performance Stratification

Patients underwent symptom-limited ETTs according to standardized protocols. The specific protocol for each test was chosen by the exercise physiologist supervising the test, based on the patient’s reported activity, and the test was performed as recommended by the exercise testing guidelines.^29^ The CRF was quantified as peak estimated METs and was determined based on treadmill grade and speed at peak exercise. Within each sex, the distribution of achieved METs separated by decade of age was used to identify the 25th, 50th, 75th, and 97.7th percentiles of CRF. Patients were then stratified a priori into performance groups by age- and sex-associated cutoffs as follows: elite (≥97.7th percentile), high (75th-97.6th percentile), above average (50th-74th percentile), below average (25th-49th percentile), and low (<25th percentile).

Estimated METs were calculated for each patient using the Veterans Affairs cohort formula for men (estimated METs = 18.7 – [0.15 × age])^30^ and the St James Take Heart Project formula for women (estimated METs = 14.7 – [0.13 × age]).^5^ In respective sexes, these formulas have been previously reported to perform best in terms of their ability to determine outcomes.^31^ Percentages of estimated METs were then calculated using the following ratio: achieved (METs/estimated METs) × 100.

### Mortality Surveillance

The primary outcome was all-cause mortality and was determined from the Social Security Death Index, when available.^32^ It was supplemented by the institutional death index (medical record documentation of patient’s death), particularly for the period following November 2011, when restrictions for the Social Security Death Index access were implemented. The final censoring date was December 31, 2017.

### Statistical Analysis

Data are reported as mean (SD) for normally distributed variables and median (interquartile range) for nonnormally distributed, continuous variables. Analysis of variance and χ^2^ testing were used for the analysis of continuous and categorical variables, respectively. The time-related all-cause mortality was analyzed using the nonparametric Kaplan-Meier method,^33^ and groups were compared using the log-rank test.

To adjust for differences in baseline characteristics between performance groups, a multivariable Cox proportional hazards regression model was constructed to obtain the risk-adjusted association between all-cause mortality and CRF. Covariates incorporated into the model included age, sex, body mass index, history of CAD, hyperlipidemia, hypertension, diabetes, smoking, ESRD, year of testing, and current use of aspirin, β-blockers, or statins. We additionally found that patients referred for ETT for specified indications other than symptoms or those related to known or suspected CAD had significantly worse survival (eFigure 1 in the Supplement). These indications for testing were incorporated into the regression model.

For all analyses, a 2-sided *P* ≤ .05 was considered statistically significant. Analyses were performed April 2018 using JMP statistical software, version 13 (SAS Institute Inc).

## Results

### Patient Demographics

A total of 122 007 patients (mean [SD] age, 53.4 [12.6] years; 72 173 [59.2%] male) were included in the final study cohort (eFigure 2 in the Supplement). Baseline characteristics are given in Table 1. The prevalence of associated comorbidities decreased significantly with increasing performance with the exception of hyperlipidemia, which was present in 31.6% (1128 of 3570) of elite performers and only 25.1% (7323 of 29 181) of low performers (*P* < .001). Temporal trends for age and comorbidities among the study cohort remained stable, although use of common cardiovascular medications increased during the study period (eFigures 3, 4, and 5 in the Supplement). Performance group cutoffs based on age and sex are given in Table 2. Evaluation for CAD and symptom assessment were the most common indications for ETT referral, with additional indications listed in the eTable in the Supplement.

**Table 1.** Patient Demographics^a^

Demographic	All Patients (N = 122 007)	Performance Group				
		Low (n = 29 181)	Below Average (n = 27 172)	Above Average (n = 31 897)	High (n = 30 187)	Elite (n = 3570)
Age, mean (SD), y	53.4 (12.6)	53.7 (12.5)	53.2 (12.7)	53.3 (12.5)	53.5 (12.6)	53.3 (12.6)
Male	72 173 (59.2)	17 496 (60.0)	15 333 (56.4)	19 040 (59.7)	18 073 (59.9)	2231 (62.5)
Maximum No. of METs, mean (SD)	9.0 (2.7)	6.1 (1.7)	8.2 (1.6)	9.6 (1.7)	11.4 (1.8)	13.8 (1.5)
Estimated METs, mean (SD), %	101.2 (27.1)	68.0 (15.4)	92.5 (8.6)	107.6 (10.3)	128.0 (15.7)	155.9 (23.5)
BMI, mean (SD)	28.7 (5.8)	31.7 (7.3)	29.8 (5.5)	28.0 (4.6)	26.2 (3.9)	24.5 (3.4)
CAD	19 197 (15.7)	6472 (22.2)	4411 (16.2)	4409 (13.8)	3551 (11.8)	354 (9.9)
CABG or PCI	10 735 (8.8)	3975 (13.6)	2393 (8.8)	2350 (7.4)	1843 (6.1)	174 (4.9)
Diabetes	14 115 (11.6)	6387 (21.9)	3537 (13.0)	2590 (8.1)	1514 (5.0)	87 (2.4)
Hypertension	53 307 (43.7)	16 820 (57.6)	12 998 (57.8)	12 693 (39.8)	9846 (32.6)	2620 (26.6)
Hyperlipidemia	32 953 (27.0)	7323 (25.1)	7114 (26.2)	8552 (26.8)	8836 (29.3)	1128 (31.6)
ESRD	1385 (1.1)	900 (3.1)	251 (0.9)	148 (0.5)	79 (0.3)	7 (0.2)
Current or prior smoker	55 577 (45.6)	16 522 (56.6)	13 292 (48.9)	13 732 (43.1)	11 014 (36.5)	1017 (28.5)
Medication use						
Aspirin	40 680 (33.3)	11 353 (38.9)	9137 (33.6)	10 055 (31.5)	9051 (30.0)	1084 (30.4)
β-Blocker	29 620 (24.3)	10 975 (37.6)	6770 (24.9)	6476 (20.3)	4957 (16.4)	442 (12.4)
Statin	32 000 (26.2)	8617 (29.5)	7177 (26.4)	7991 (25.1)	7360 (24.4)	855 (24.0)
Follow-up, median (IQR), y	8.4 (4.3-13.4)	7.9 (3.8-13.1)	9.0 (4.5-14.2)	8.9 (4.6-14.1)	8.2 (4.3-12.8)	7.1 (3.8-10.7)
Death (all-cause)	13 637 (11.2)	6904 (23.7)	2888 (10.6)	2340 (7.3)	1412 (4.7)	93 (2.6)

Abbreviations: BMI, body mass index (calculated as weight in kilograms divided by height in meters squared); CABG, coronary artery bypass grafting; CAD, coronary artery disease; ESRD, end-stage renal disease; IQR, interquartile range; METs, metabolic equivalents; PCI, percutaneous coronary intervention.

^a^ Data are presented as number (percentage) of patients unless otherwise indicated. *P* < .001 for all categories.

**Table 2.** Classification of Cardiorespiratory Fitness by Age and Sex^a^

Age, y	Performance Group				
	Low	Below Average	Above Average	High	Elite
Women					
18-19	<10.0	10-11.0	11.1-12.9	13-14.9	≥15.0
20-29	<8.0	8.0-9.9	10-11.4	11.5-14.2	≥14.3
30-39	<7.7	7.7-9.3	9.4-10.8	10.9-13.6	≥13.7
40-49	<7.4	7.4-8.9	9.0-10.3	10.4-13.2	≥13.3
50-59	<7.0	7.0-8.0	8.1-9.9	10.0-12.9	≥13.0
60-69	<6.0	6.0-6.9	7.0-8.4	8.5-11.0	≥11.1
70-79	<5.0	5.0-5.9	6.0-6.9	7.0-9.9	≥10.0
≥80	<4.4	4.4-5.4	5.5-6.2	6.3-8.3	≥8.4
Men					
18-19	<10.8	10.8-12.9	13.0-13.9	14-16.2	≥16.3
20-29	<10.3	10.3-11.9	12.0-13.6	13.7-15.6	≥15.7
30-39	<10.0	10-11.1	11.2-12.9	13.0-14.9	≥15.0
40-49	<9.8	9.8-10.9	11.0-12.4	12.5-14.6	≥14.7
50-59	<8.2	8.2-9.9	10.0-11.3	11.4-13.9	≥14.0
60-69	<7.0	7.0-8.4	8.5-9.9	10.0-12.9	≥13.0
70-79	<6.0	6.0-6.9	7.0-8.4	8.5-11.4	≥11.5
≥80	<5.1	5.1-6.2	6.3-7.2	7.3-9.9	≥10.0

^a^ Ranges are given in metabolic equivalents, with 1 metabolic equivalent equaling 3.5 mL/kg per minute of oxygen consumption. Classification (percentile range) is as follows: low (<25th percentile), below average (25th-49th percentile), above average (50th-74th percentile), high (75th-97.6th percentile), and elite (≥97.7th percentile).

### Survival Analysis

**Figure 1.**
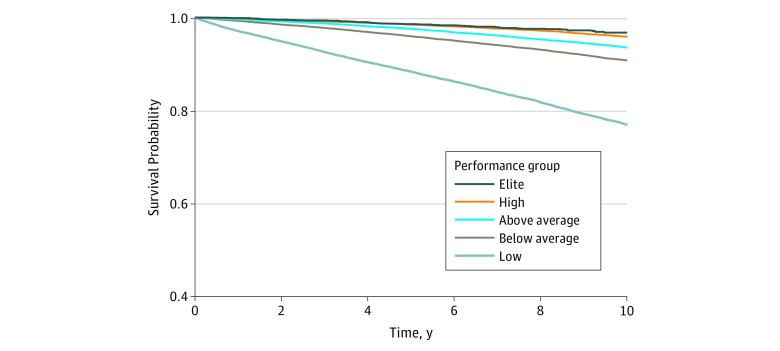
Patient Survival by Performance Group Log-rank *P* < .001 for all groups, except elite vs high performers (log-rank *P* = .002). Performance group classifications by cardiorespiratory fitness are defined in Table 2.

Death from any cause occurred in 13 637 patients during a median follow-up of 8.4 years (range, 4.3-13.4 years) and 1.1 million person-years of observation. Kaplan-Meier curves of all-cause mortality stratified by performance groups demonstrated significant, incremental reduction in all-cause mortality associated with increasing performance (Figure 1). Elite performers had increased unadjusted survival compared with all other groups, including high performers.

To assess for potential confounding related to indications for ETT, survival analysis was performed by referral indications. Indications were grouped into 3 categories: symptoms only, known or suspected CAD, or other or non-CAD indications. Kaplan-Meier curves by indication groups demonstrated significantly worse survival in patients referred for other or non-CAD indications for ETT (eFigure 1 in the Supplement).

### Cox Proportional Hazards Regression

**Figure 2.**
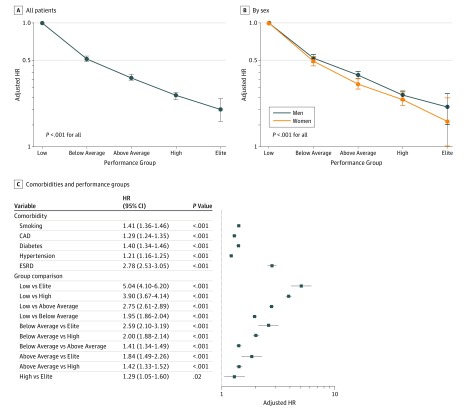
Risk-Adjusted All-Cause Mortality Adjusted hazard ratios (HRs) for all-cause mortality compared with low performers in all patients (A) and by sex (B) (*P* values are for comparisons with low performers). C, Adjusted HRs for comorbidities and between performance groups. Error bars indicate 95% CIs. Performance group classifications by cardiorespiratory fitness are defined in Table 2. CAD indicates coronary artery disease; and ESRD, end-stage renal disease.

After multivariate adjustment, incremental reduction in all-cause mortality was associated with increasing performance when compared with low performers (Figure 2A). Similar findings were observed when separated by sex (Figure 2B). Adjusted hazard ratios (HRs) for clinical comorbidities and among performance groups are shown in Figure 2C. Risk-adjusted all-cause mortality was inversely proportional to CRF and was lowest in elite performers (elite vs low: adjusted HR, 0.20; 95% CI, 0.16-0.24; *P* < .001). The increase in all-cause mortality associated with reduced CRF (low vs elite: adjusted HR, 5.04; 95% CI, 4.10-6.20; *P* < .001; below average vs above average: adjusted HR, 1.41; 95% CI, 1.34-1.49; *P* < .001) was comparable to or greater than traditional clinical risk factors. Adjusted mortality risk was expectedly highest in the lowest performing groups and generally exceeded that of traditional clinical risk factors, including CAD (adjusted HR, 1.29; 95% CI, 1.24-1.35; *P* < .001), smoking (adjusted HR, 1.41; 95% CI, 1.36-1.46; *P* < .001), and diabetes (adjusted HR, 1.40; 95% CI, 1.34-1.46; *P* < .001). Multivariate Cox proportional hazards regression with less stringent (≥95th percentile) and more stringent (≥99th percentile) elite performance cutoffs demonstrated consistent findings (eFigure 6 in the Supplement).

**Figure 3.**
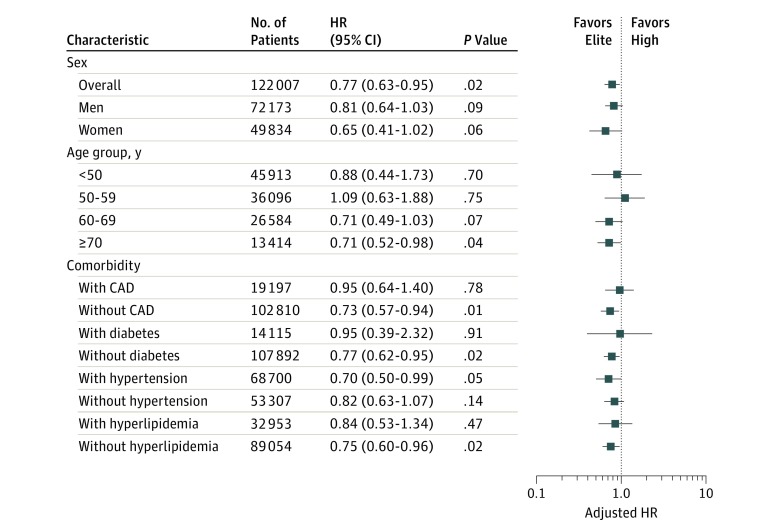
Adjusted Mortality Risk in Elite vs High Performers Multivariable adjusted Cox proportional hazards regression model for specified subgroups. Adjusted hazard ratios (HRs) are for elite vs high performers. Error bars indicate 95% CIs. CAD indicates coronary artery disease. Performance group classifications by cardiorespiratory fitness are defined in Table 2.

Multivariate analyses also demonstrated improved survival in elite vs high performers (adjusted HR, 0.77; 95% CI, 0.63-0.95; *P* = .02). By sex, a nonstatistically significant improved survival in elite vs high performers was present in both men and women (men: adjusted HR, 0.81; 95% CI, 0.64-1.03; *P* = .09; women: adjusted HR, 0.65; 95% CI, 0.41-1.02; *P* = .06) (Figure 3). When analyzed by age group, the difference in survival between elite and high performers was only maintained in older patients. There was a statistically significant reduction in mortality between elite and high performers 70 years or older (adjusted HR, 0.71; 95% CI, 0.52-0.98; *P* = .04). In younger age groups, there was no difference in survival between elite and high performers.

Further multivariate regression was performed in the following clinical subgroups: known CAD, diabetes, hypertension, and hyperlipidemia. In all comorbid subgroups, all-cause mortality was inversely proportional to CRF and lowest in elite performers (eFigure 7 in the Supplement). In patients with hypertension, elite performers had a significant reduction in risk-adjusted all-cause mortality compared with high performers (adjusted HR, 0.70; 95% CI, 0.50-0.99; *P* = .05). In all other comorbid subgroups, there was no statistical difference in survival between elite and high performers.

## Discussion

We report long-term mortality data from the largest cohort of patients undergoing ETT at a tertiary care center (n = 122 007; 1.1 million patient-years of observation). As quantified by peak estimated METs on ETT, CRF was significantly and inversely associated with all-cause mortality. The adjusted mortality risk of reduced performance on ETT was comparable to, if not greater than, traditional clinical risk factors (eg, CAD, smoking). Importantly, there was no upper limit of benefit of increased aerobic fitness. Elite performers (CRF ≥2 SDs above the mean for age and sex) had an incremental reduction in all-cause mortality compared with all other performance groups, including high performers (75th-97.6th percentile of age- and sex-matched CRF). In subgroup analysis, the survival benefit of elite vs high performers was present in older adults and those with hypertension. These findings emphasize the importance of aerobic fitness in overall health, including the magnitude of benefit of increased CRF in relation to traditional clinical risk factors and the incremental survival advantage of extremely high fitness.

Previous studies^1,2,3,4,5,6^ have consistently demonstrated a reduction in mortality associated with higher levels of aerobic fitness. The present study reinforces these findings with data from a large cohort of patients referred for ETT. Even after adjustment for baseline clinical characteristics, the magnitude of benefit of increased aerobic fitness remains particularly striking. When compared with the lowest performers, elite performance was associated with an 80% reduction in mortality risk. In addition, the adjusted mortality risk of reduced performance was comparable to, if not significantly greater than, traditional clinical risk factors, such as CAD, diabetes, and smoking (Figure 2C). This finding remains true even when comparing adjacent performance groups; the reduction in mortality risk was seen in a dose-effect manner with any increment in CRF. These findings not only reinforce the large collective body of evidence correlating aerobic fitness with numerous health benefits but also illustrate the importance of aerobic fitness as a powerful, modifiable indicator of long-term mortality.

There continues to be uncertainty regarding the relative benefit or potential risk of extreme levels of exercise and fitness. Significant attention has been paid to better understand the long-term cardiovascular effects of extreme exercise.^34^ Potentially adverse cardiovascular findings in highly active cohorts, including an increased incidence of atrial fibrillation, coronary artery calcification, myocardial fibrosis, and aortic dilation, have raised concern for potential cardiovascular risk above a certain exercise or training threshold. It remains unclear whether these associations are signals of true pathologic findings or rather benign features of cardiovascular adaptation. The present study is the first, to our knowledge, to specifically evaluate the association between extremely high CRF and long-term mortality.

We found that elite performers undergoing ETT had a significant association with reduction in all-cause mortality when compared with any other performance group. Overall, increases in CRF were associated with a reduction in all-cause mortality at any level, without evidence of a plateau effect or U-shaped association (Figure 2A). There does not appear to be an upper limit of aerobic fitness above which a survival benefit is no longer observed. These results are in concordance with previous observational studies^35,36,37,38^ of highly active cohorts and other large, longitudinal studies^4,6^ of CRF and mortality but are notably discrepant from population-based studies^24,25,26^ of physical activity and exercise. This difference may reflect the objective measurement of physical fitness in the present study, as opposed to self-reported activity levels, which have been a major limitation of prior studies.^24,25,26^ It may also reflect non–activity-related contributors to aerobic fitness, including genetic factors and unmeasured health habits, which may contribute to improved survival.^35,39,40^ Lastly, it may, in part, be attributable to differences in the observed populations in that patients referred for ETT are distinct from the general population, with presumably higher incidences of clinical pathologic findings that influence fitness. Regardless of these discrepancies, in patients referred for ETT, it is evident that higher CRF, even to extreme levels, is associated with improved survival.

Achieving and maintaining very high levels of aerobic fitness may be particularly important in older patients (≥70 years of age) and those with hypertension. Elite performance was significantly associated with improved survival in these groups compared with high performers. An age-associated benefit of CRF has been demonstrated previously in older adults,^1^ though without specific attention to extremely high levels of fitness. Extremely high CRF in older patients is likely to reflect long-term activity and/or exercise habits, and the cumulative benefits of high aerobic fitness may contribute to a more significant effect on long-term survival. Older patients may also derive additional benefits outside those traditionally ascribed to CRF, including reductions in overall frailty and maintenance of physical independence. Furthermore, this finding may reflect unique selection factors present in older patients who are able to continue with such high levels of activity. Clinically, this age-related association is significant because it emphasizes the importance of continued physical activity in older adults and the benefits of extremely high levels of fitness in elderly people. The benefit of elite performance in patients with hypertension is consistent with previous data showing CRF to be the strongest factor associated with survival in these patients, more so than any other clinical risk factors.^9^ It also reinforces guideline recommendations for lifestyle modifications, including physical activity and exercise, in all patients with hypertension.^39^

The relative benefit of extremely high fitness may be attenuated in patients with known CAD or certain cardiac risk factors. No statistically significant difference was found in all-cause mortality between elite and high performers with established CAD, hyperlipidemia, or diabetes. The number of elite performers with these comorbidities was comparatively small and may have limited the statistical power to detect an associated mortality difference. Overall, higher CRF was associated with improved survival in these subgroups (eFigure 7 in the Supplement). The rates of cardiac and noncardiac comorbidities (except hyperlipidemia) were significantly lower in elite performers compared with all other performance groups. There was also no evidence to suggest relative harm associated with extreme levels of fitness in these subsets of patients. Collectively, these data demonstrate improved survival associated with increased CRF in patients with known CAD or certain cardiac comorbidities, without evidence of relative harm at extreme levels of fitness.

A significant increase was found in unadjusted mortality in patients referred for ETT for other or non-CAD indications. The most common indication in this subgroup was evaluation of valvular disease, which has been a practice at our institution since the 1990s. The observed reduction in survival in this group may be driven by unique clinical factors and mortality risk among patients with severe valvular disease.

### Limitations

The primary limitation of the study reflects its retrospective nature, in that the association between CRF and mortality does not prove causation. The degree to which high CRF preselects patients with lower mortality vs causes a reduction in mortality is not discernible from our study. In addition, although we account for many clinical factors, there may be confounding variables that are unaccounted for in our analyses, including socioeconomic status, race/ethnicity, and others. These limitations apply to other studies of aerobic fitness and long-term outcomes and are unlikely to be overcome because of the infeasibility of a large-scale, randomized study. Given these inherent limitations, our study demonstrates that patients who can exercise to an extreme level live longest, acknowledging that there may be many measured and unmeasured factors that contribute to this association.

We also recognize that the study population (ie, those referred for ETT) may not reflect the general population distribution of estimated functional capacity for the purpose of identifying elite performers. However, there is no widely accepted standard for classification of CRF based on ETT performance. Recent proposals have suggested similar age-specific methods for CRF standardization.^40^ The method outlined in the present study adequately defines a cohort of patients with exceptionally high CRF suitable to test the underlying hypothesis. With the use of previously validated models to estimate functional capacity in adults,^5,30^ the elite performance group in this study had a mean estimated functional capacity 55% greater than estimated by age and sex. Cutoff values for elite performers were consistent with historic normative data,^41^ and measured CRF of elite performers was comparable to published age- and sex-matched cohorts of endurance athletes^42,43,44^

Cardiorespiratory fitness was based on a patient’s performance on a single ETT, and these findings do not speak to the association between long-term levels of fitness and mortality. However, this study reinforces the clinical utility of using ETT to better understand and determine patient prognosis. Even measured at a single time point, performance on ETT is remarkably correlated with long-term survival.

## Conclusions

Increased CRF was associated with reduced long-term mortality with no observed upper limit of benefit. The adjusted mortality risk of reduced CRF was greater than or equal to traditional clinical risk factors, such as cardiovascular disease, diabetes, and smoking. Extreme aerobic fitness (CRF ≥2 SDs above the mean for age and sex) was associated with the greatest survival and was notably beneficial in older patients and those with hypertension. Cardiorespiratory fitness is a modifiable indicator of long-term mortality, and health care professionals should encourage patients to achieve and maintain high levels of fitness.

## Notes

Supplement. **eMethods.** Definitions of Comorbid Conditions **eFigure 1.** Kaplan-Meier Curves by Indication for Exercise Treadmill Testing **eFigure 2.** Study Cohort Flow Chart **eFigure 3.** Mean Age of the Cohort by Year of Study **eFigure 4.** Trends in Male Referral and Comorbidities by Year of Study **eFigure 5.** Trends in Medication Use by Year of Exercise Treadmill Testing **eFigure 6.** Risk-Adjusted All-Cause Mortality by Elite Performance Cutoff **eFigure 7.** Adjusted Risk of Death in Patients With Comorbidities **eTable.** Most Common Indications for Exercise Stress Testing
