# Supplementary material for: Association of Cardiorespiratory Fitness With Long-term Mortality Among Adults Undergoing Exercise Treadmill Testing
Source: JAMA Netw Open. 2018 Oct 19;1(6):e183605. doi: 10.1001/jamanetworkopen.2018.3605 (PMC6324439; doi:10.1001/jamanetworkopen.2018.3605)
Supplement: Supplement. — eMethods. Definitions of Comorbid Conditions eFigure 1. Kaplan-Meier Curves by Indication for Exercise Treadmill Testing eFigure 2. Study Cohort Flow Chart eFigure 3. Mean Age of the Cohort by Year of Study eFigure 4. Trends in Male Referral and Comorbidities by Year of Study eFigure 5. Trends in Medication Use by Year of Exercise Treadmill Testing eFigure 6. Risk-Adjusted All-Cause Mortality by Elite Performance Cutoff eFigure 7. Adjusted Risk of Death in Patients With Comorbidities eTable. Most Common Indications for Exercise Stress Testing [file jamanetwopen-1-e183605-s001.pdf]

Supplementary Online Content

Mandsager K, Harb S, Cremer P, Phelan D, Nissen SE, Jaber W. Association of cardiorespiratory fitness with long-term mortality among adults undergoing exercise treadmill testing. *JAMA Netw Open*. 2018;1(6):e20183605.

eMethods. Definitions of Comorbid Conditions

eFigure 1. Kaplan-Meier Curves by Indication for Exercise Treadmill Testing

eFigure 2. Study Cohort Flow Chart

eFigure 3. Mean Age of the Cohort by Year of Study

eFigure 4. Trends in Male Referral and Comorbidities by Year of Study

eFigure 5. Trends in Medication Use by Year of Exercise Treadmill Testing

eFigure 6. Risk-Adjusted All-Cause Mortality by Elite Performance Cutoff

eFigure 7. Adjusted Risk of Death in Patients With Comorbidities

eTable. Most Common Indications for Exercise Stress Testing

This supplementary material has been provided by the authors to give readers additional information about their work.

© 2018 Mandsager K et al. *JAMA Network Open.*

eMethods. Definitions of Comorbid Conditions

Coronary Artery Disease: prior myocardial infarction or prior coronary revascularization

Diabetes Mellitus: self-reported history or use of glucose-lowering medications

Hypertension: self-reported history or use of anti-hypertensive medications (angiotensin converting enzyme inhibitors, angiotensin receptor blockers, calcium channel blockers, or thiazide diuretics)

Hyperlipidemia: Abnormal lipid panel (meeting accepted diagnostic criteria at the time of treadmill testing) or use of statin medications

Smoker: current or prior tobacco use

End-stage renal disease: receiving dialysis

© 2018 Mandsager K et al. *JAMA Network Open.*

eFigure 1. Kaplan-Meier Curves by Indication for Exercise Treadmill Testing

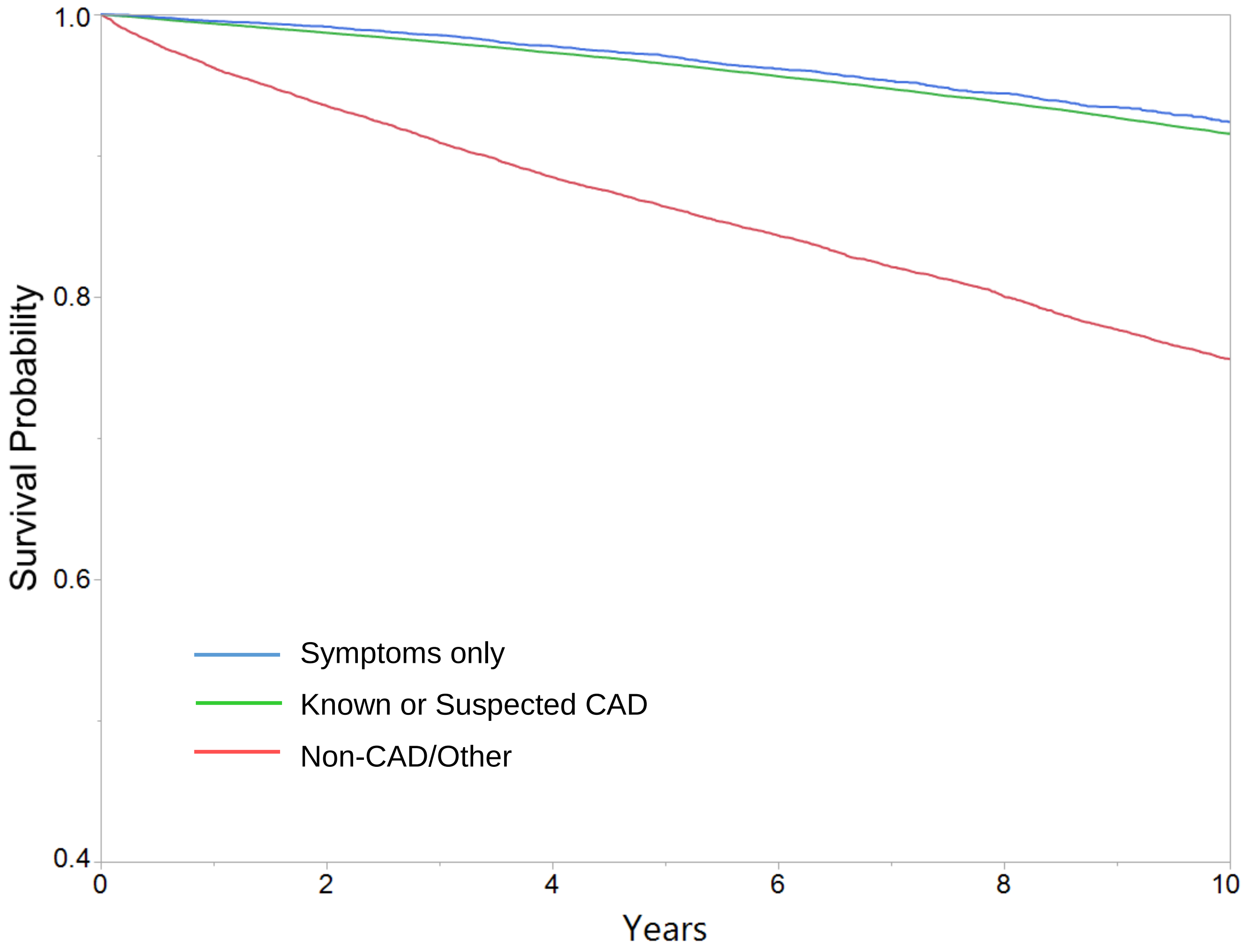

Log rank *p*<0.0001. CAD = coronary artery disease

© 2018 Mandsager K et al. *JAMA Network Open.*

eFigure 2. Study Cohort Flow Chart

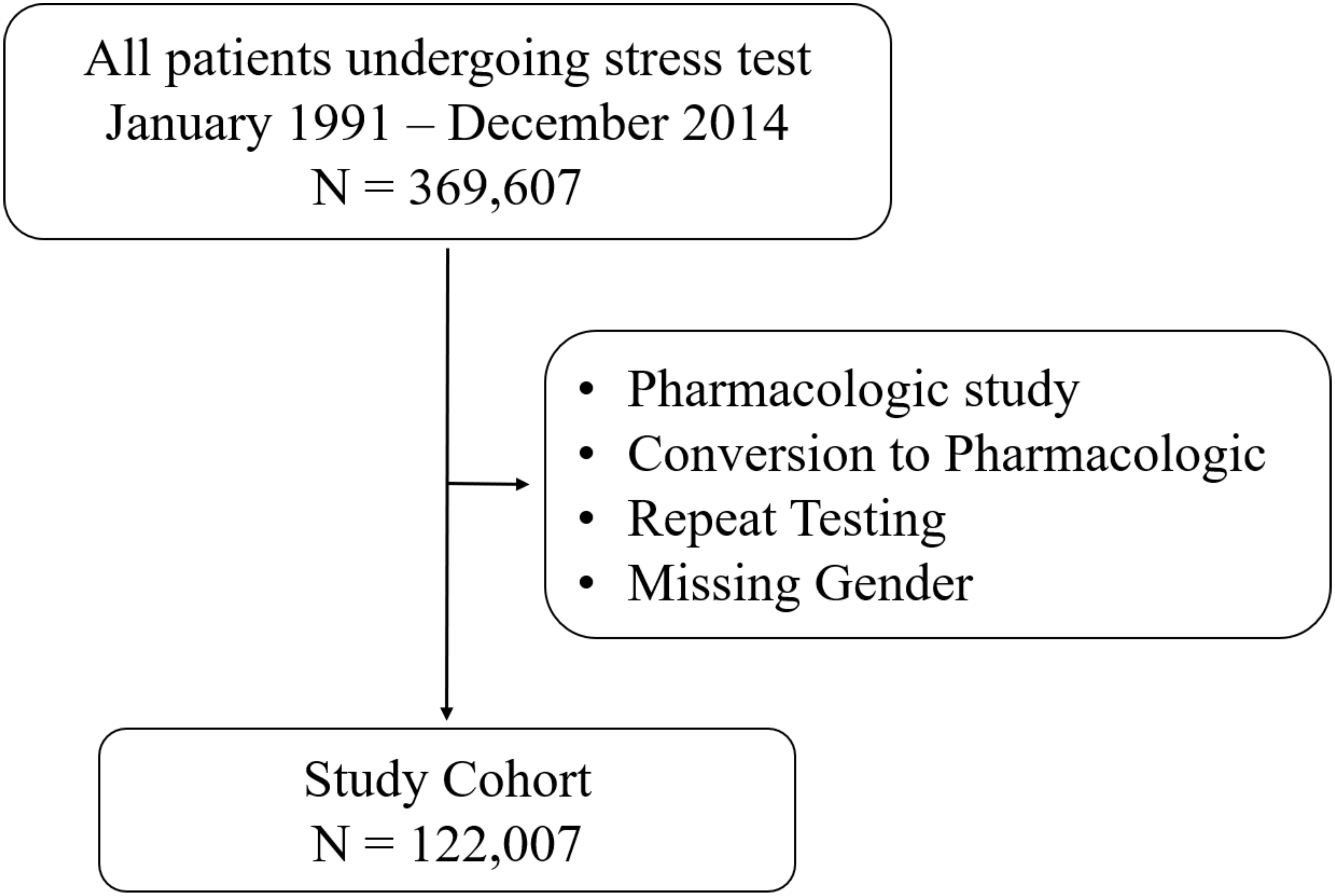

© 2018 Mandsager K et al. *JAMA Network Open.*

eFigure 3. Mean Age of the Cohort by Year of Study

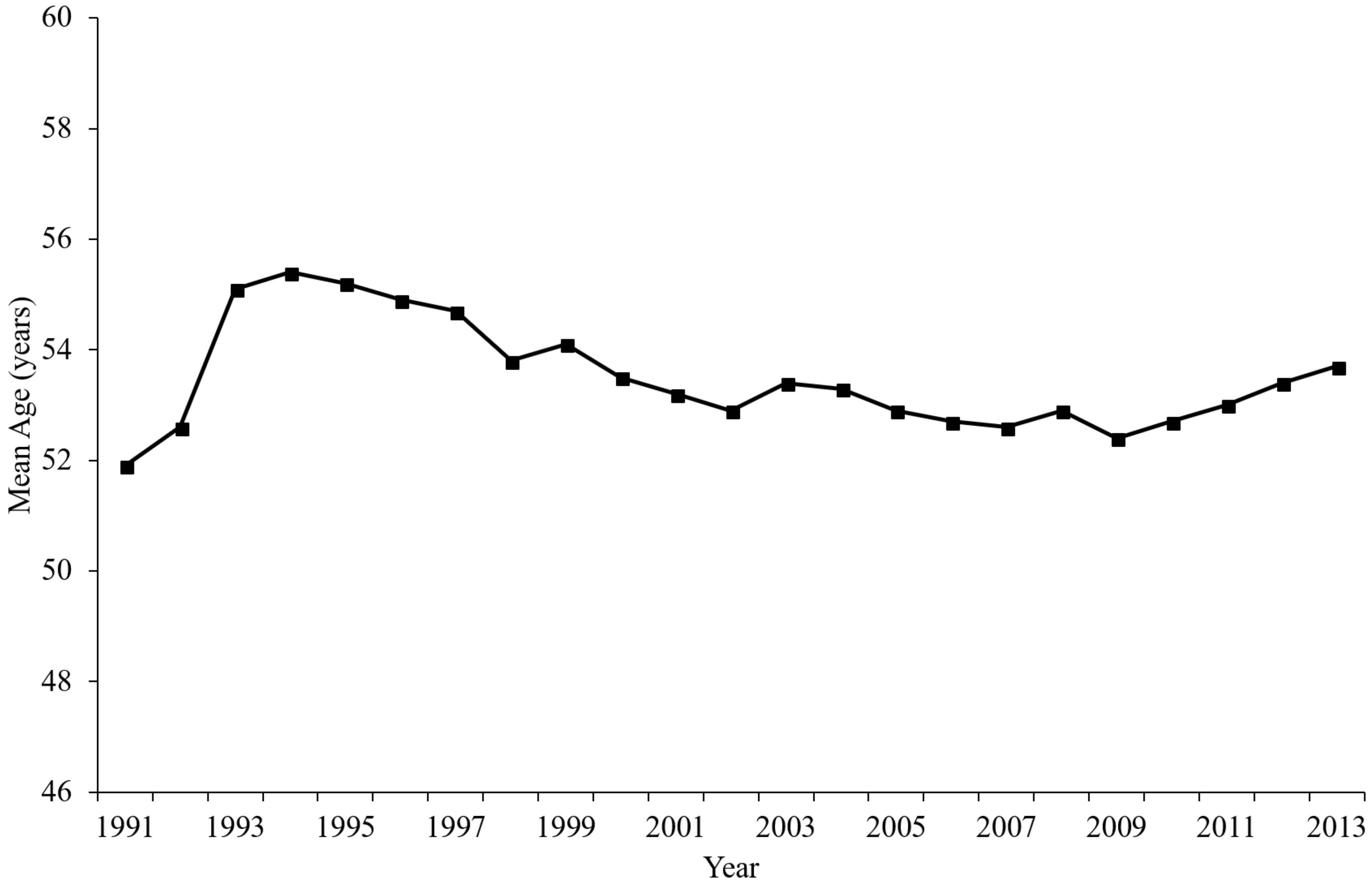

© 2018 Mandsager K et al. *JAMA Network Open.*

eFigure 4. Trends in Male Referral and Comorbidities by Year of Study

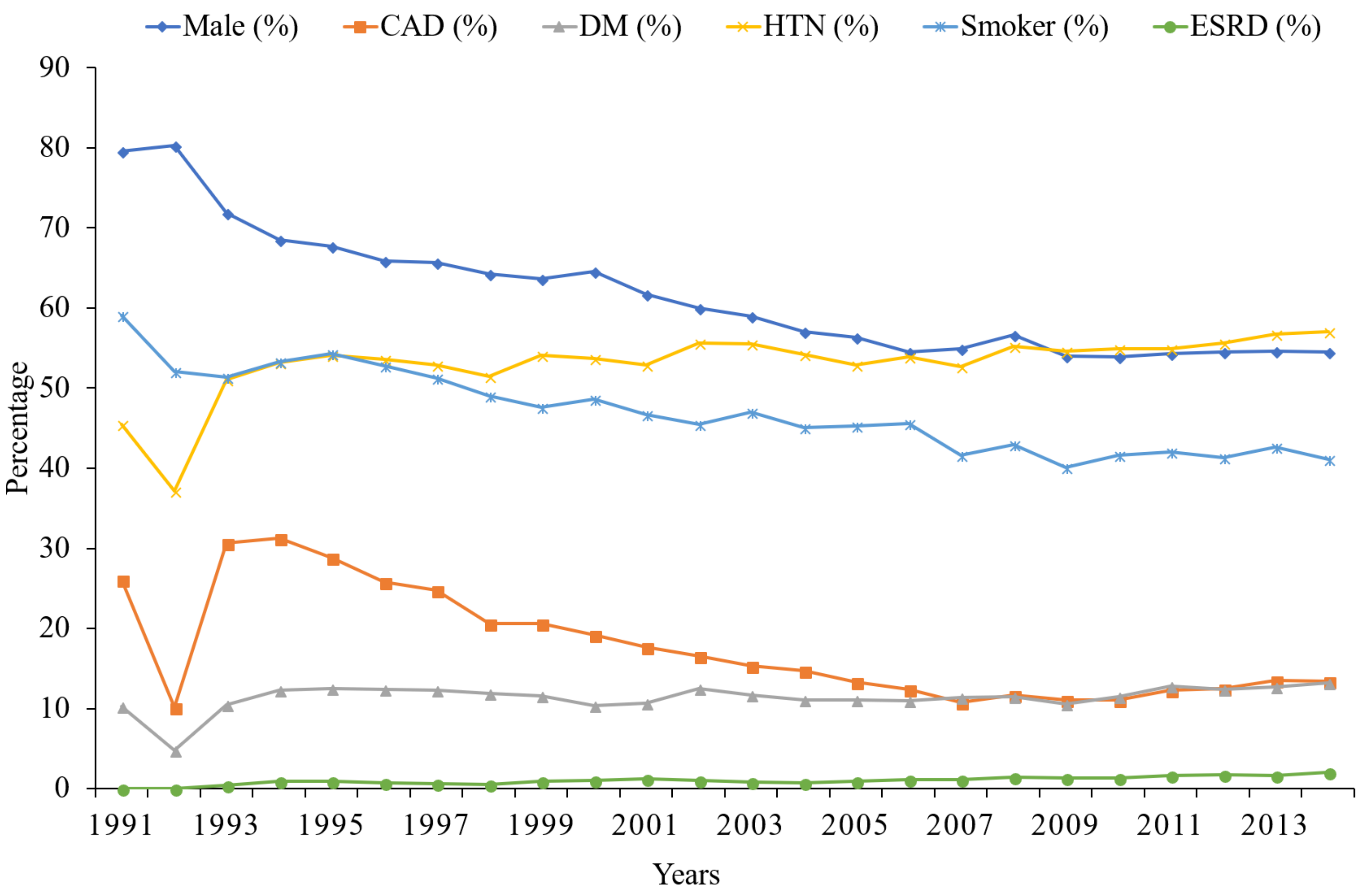

CAD = coronary artery disease; DM = Diabetes Mellitus; HTN = Hypertension; ESRD = end-stage renal disease.

© 2018 Mandsager K et al. *JAMA Network Open.*

eFigure 5. Trends in Medication Use by Year of Exercise Treadmill Testing

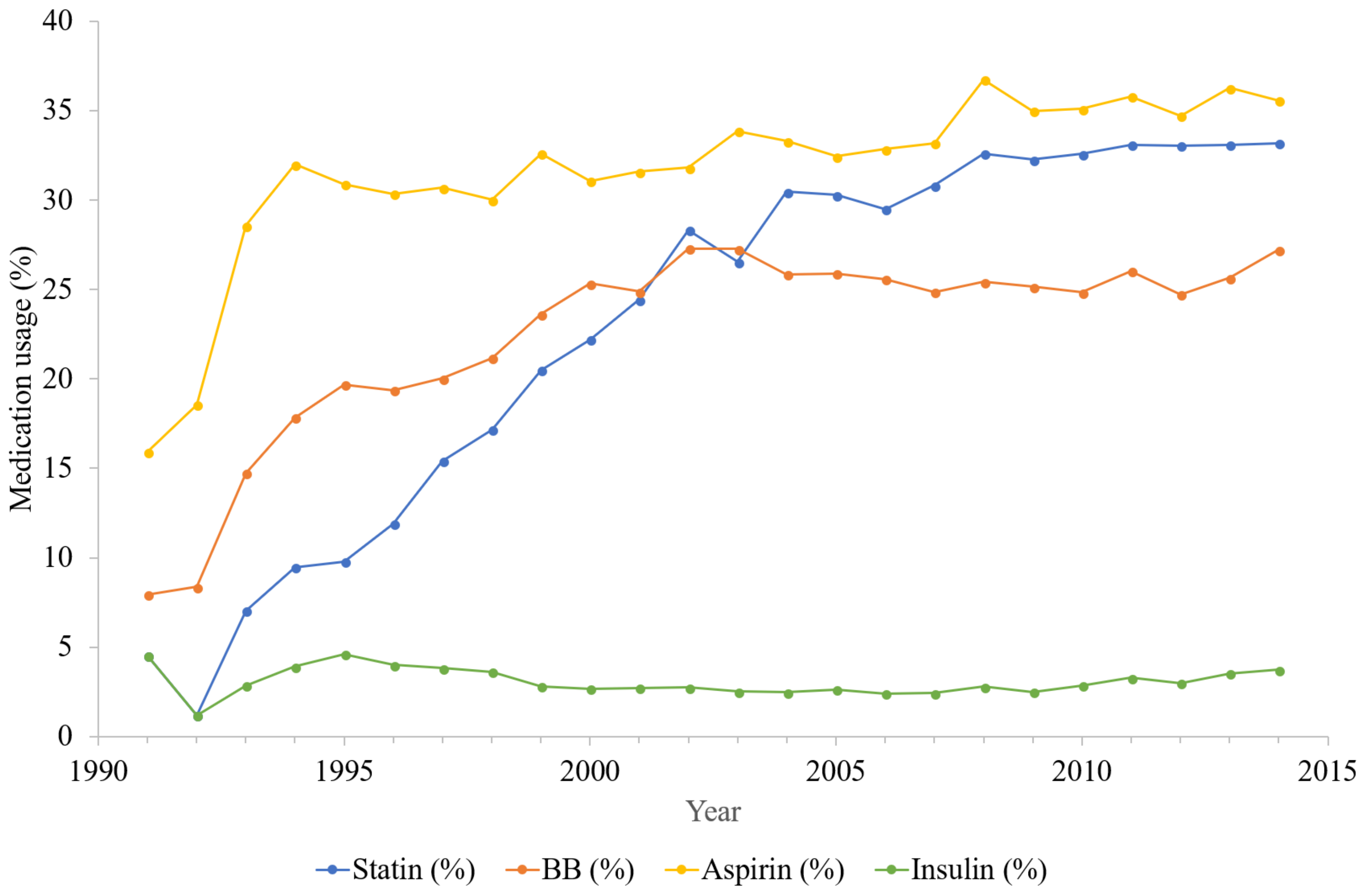

BB = Beta-blocker

© 2018 Mandsager K et al. *JAMA Network Open.*

eFigure 6. Risk-Adjusted All-Cause Mortality by Elite Performance Cutoff

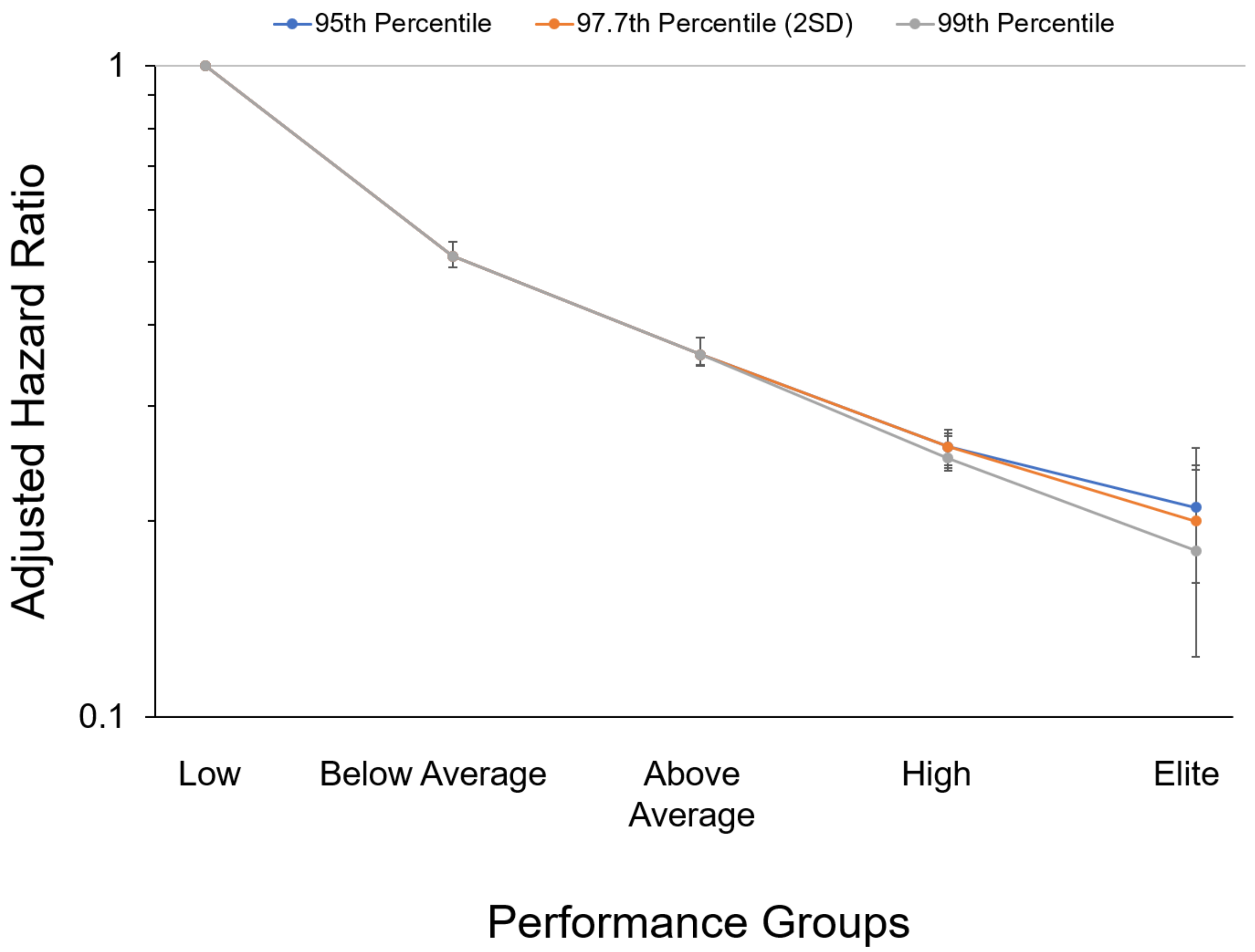

© 2018 Mandsager K et al. *JAMA Network Open.*

eFigure 7. Adjusted Risk of Death in Patients With Comorbidities

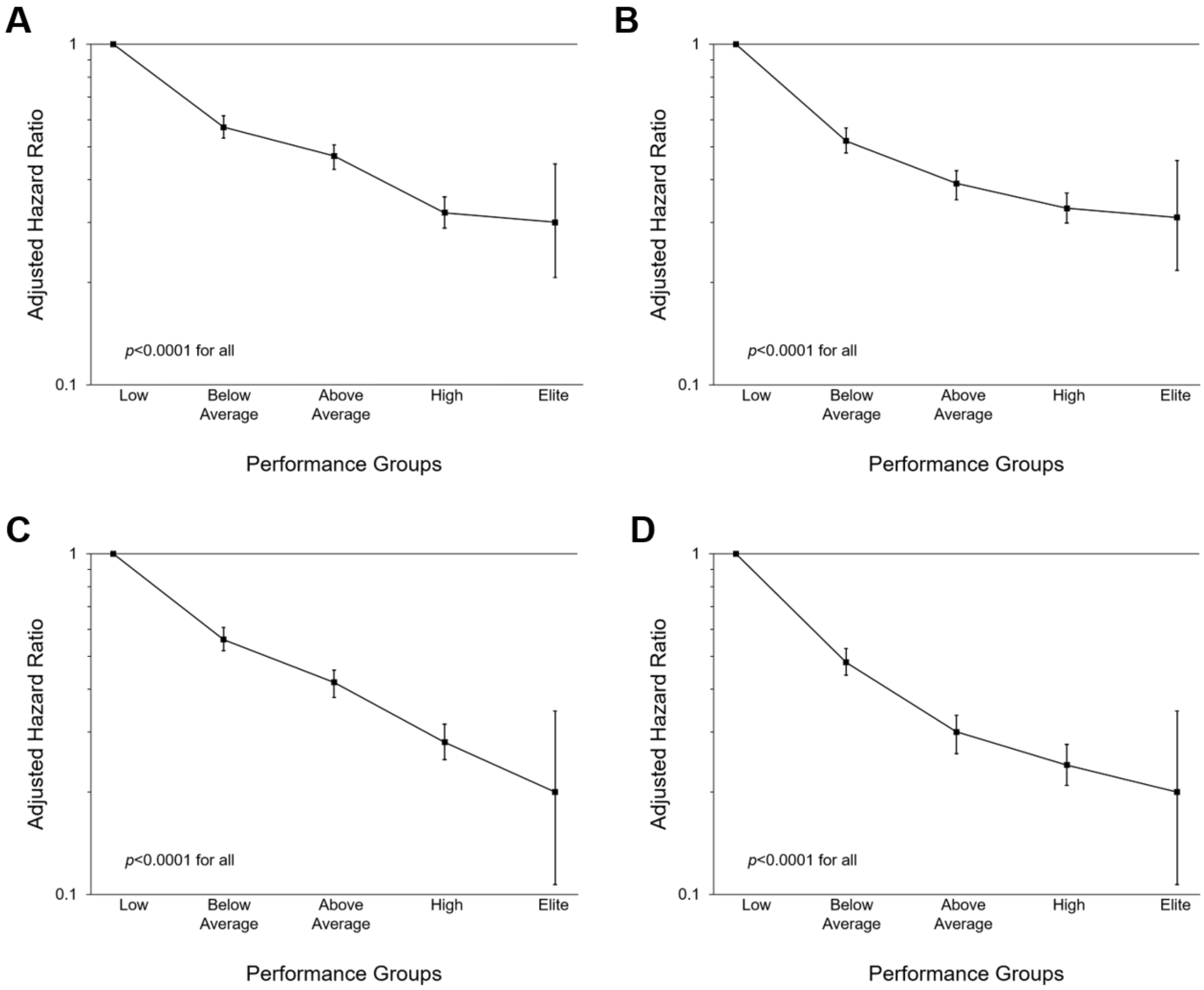

Patients with **(A)** coronary artery disease; **(B)** diabetes; **(C)** hypertension; **(D)** hyperlipidemia. *p*-values compared to low performers.

© 2018 Mandsager K et al. *JAMA Network Open.*

eTable. Most Common Indications for Exercise Stress Testing

Indication for exercise stress testing	No. (%)
Rule out CAD	80,197 (65.8)
Symptom evaluation	62,461 (51.2)
Risk factors for CAD	20,507 (16.8)
Follow-up known CAD	15,941 (13.0)
Others*	15,474 (12.7)

Indications are not mutually exclusive. *Others with ≥ 4%: preoperative clearance (4%), arrhythmia evaluation (5%) and valvular disease (8%).

CAD = coronary artery disease.

© 2018 Mandsager K et al. *JAMA Network Open.*
